# Community-based bilingual doula support during labour and birth to improve migrant women’s intrapartum care experiences and emotional well-being–Findings from a randomised controlled trial in Stockholm, Sweden [NCT03461640]

**DOI:** 10.1371/journal.pone.0277533

**Published:** 2022-11-18

**Authors:** Erica Schytt, Anna Wahlberg, Amani Eltayb, Nataliia Tsekhmestruk, Rhonda Small, Helena Lindgren

**Affiliations:** 1 Division of Reproductive Health, Department of Women’s and Children’s Health, Karolinska Institutet, Stockholm, Sweden; 2 Centre for Clinical Research Dalarna, Uppsala University, Falun, Sweden; 3 Faculty of Health and Social Sciences, Western Norway University of Applied Sciences Norway, Bergen, Norway; 4 La Trobe University, Judith Lumley Centre, Melbourne Victoria, Australia; Norwegian Refugee Council, JORDAN

## Abstract

**Objectives:**

To evaluate the effectiveness of community-based bilingual doula (CBD) support for improving the intrapartum care experiences and postnatal wellbeing of migrant women giving birth in Sweden.

**Design:**

Randomised controlled trial.

**Setting:**

Six antenatal care clinics and five hospitals in Stockholm, Sweden.

**Participants:**

164 pregnant Somali-, Arabic-, Polish-, Russian- and Tigrinya-speaking women who could not communicate fluently in Swedish, were ≥18 years and had no contra-indications for vaginal birth.

**Intervention:**

In addition to standard labour support, women were randomised to CBD support (n = 88) or no such support during labour (n = 76). Trained CBDs met with women prior to labour, provided support by telephone after labour had started, then provided emotional, physical and communication support to women throughout labour and birth in hospital, and then met again with women after the birth.

**Primary outcomes:**

Women’s overall ratings of the intrapartum care experiences (key question from the Migrant Friendly Maternity Care Questionnaire) and postnatal wellbeing (mean value of Edinburgh Postnatal Depression Scale) at 6–8 weeks after birth.

**Results:**

In total, 150 women remained to follow-up; 82 women (93.2%) randomised to receive CBD support and 68 women (89.5%) randomised to standard care (SC). Of women allocated CBD support, 60 (73.2%) received support during labour. There were no differences between the groups regarding women’s intrapartum care experiences (very happy with care: CBD 80.2% (n = 65) *vs* SC 79.1% (n = 53); OR 1.07 CI 95% 0.48–2.40) or emotional wellbeing (EPDS mean value: CBD 4.71 (SD 4.96) *vs* SC 3.38 (SD 3.58); mean difference 1.33; CI 95% - 0.10–2.75).

**Conclusions:**

Community-based doula support during labour and birth for migrant women neither increased women’s ratings of their care for labour and birth nor their emotional well-being 2 months postpartum compared with receiving standard care only. Further studies on the effectiveness of CBD powered to evaluate obstetric outcomes are needed.

**Trial registration:**

**Trial registration** at ClinicalTrial.gov NCT03461640 https://www.google.com/search?client=firefox-b-d&q=NCT03461640.

## Introduction

The increasing proportion of births to migrant women in Sweden [1] and in other high-income countries requires attention to adapt care for the needs of a diverse population. Worldwide, many groups of migrant women carry a higher risk of adverse pregnancy outcomes [2,3], such as very preterm birth and stillbirth [4–6], ‘near-miss’ morbidity [7] as well as obstetric interventions, such as induction of labour and emergency caesarean section, compared with non-migrant women [2,8,9]. Barriers to equitable and high-quality care for migrant women are communication difficulties, lack of familiarity with how care is provided, and prejudicial staff attitudes and discrimination [10]. Migrant women also report being left alone, feeling fearful, unsafe and unsupported [11,12].

Community-Based Doulas (CBDs) are bilingual women from migrant communities who are trained in childbirth and labour support, and who facilitate communication between the woman/partner and staff during labour and birth [13]. Previous studies support the potential for CBDs to enhance migrant women’s experiences of birth and of care, their pregnancy outcomes and postpartum well-being. The Cochrane systematic review of 22 trials evaluating continuous support for women during childbirth [14] found greater maternal satisfaction with care, less use of analgesia, shorter labours, lower rates of caesarean section and more spontaneous vaginal births–with no adverse effects for women or infants. The review concluded that all women should have someone with them throughout labour and also suggested this was most effective when support was provided by an outsider, not a hospital staff member or someone from women’s own social networks [14]. One descriptive US study showed that Somali women who were provided with a hospital-based doula had greater satisfaction with care and lower rates of caesarean section than women without such support [15]. Another US cohort study (n = 11 471) that evaluated a community doula program in a culturally diverse setting found a small reduction in caesarean section among women cared for by a midwife and a doula, compared with a midwife alone (15% *vs* 18%)[16]. Further, a Cochrane qualitative evidence synthesis of 23 studies showed that foreign-born women in high-income countries appreciate support from community-based doulas to receive culturally-competent care [17]. Qualitative studies also show mainly positive views about doula support to women in labour among midwives and obstetricians who reported that CBDs contribute communication assistance and help to improve safety during labour and birth [18–20]. Yet, no randomised controlled trials evaluating the effectiveness of CBD support have been undertaken.

There is a growing interest worldwide in implementing CBDs to provide support for migrant women during labour and birth, but more robust and specific evidence is needed to help policy makers make decisions on whether the CBD model should be implemented universally for migrant women, or not. This is the first randomised controlled trial to evaluate the effectiveness of CBD support for improving the intrapartum care experiences and postnatal wellbeing of specific groups of migrant women: Arabic-, Polish-, Russian-, Somali-, and Tigrinya-speaking migrant women giving birth in Stockholm, Sweden. We hypothesised that migrant women randomised to receive CBD support in labour, in addition to standard labour support, would 1) rate their care for labour and birth more highly, and 2) experience better emotional wellbeing (lower mean scores on the Edinburgh Postnatal Depression Scale) two months after the birth, than migrant women allocated to standard care (SC).

## Methods

This study was a pragmatic randomised controlled trial (RCT) conducted between 28 February 2018 and 15 March 2020, with final data collection completed on 15 May 2020. The trial was closed at the outbreak of the Covid-19 pandemic when only one support person was allowed in the labour ward. The study protocol [13] describes in detail the study setting and design, rationale, program theory and methods. The trial follows the Standard Protocol Items: Recommendation for Interventional Trials statement [21] and is registered at ClinicalTrials.gov (NCT03461640). The women were informed that 1) participation in the study was voluntary, 2) their decision whether or not to participate would not affect their current or future treatment, 3) if they decided to participate they were free to withdraw at any time, and 4) all questionnaire data were de-identified. The women who agreed to participate in the study gave their verbal informed consent to participate. The study was approved by the Regional Ethical Review Board in Stockholm (approval number: 2018/12–31/2).

### Context for implementation

Midwives and obstetricians in Stockholm labour wards and ANC clinics had been advocating for some time for extra labour support for migrant women. A political decision to introduce CBDs, replicating a model developed, refined and successfully implemented by midwives over several years in Gothenburg (see below), was made by Stockholm Region in 2016. Funding was made available to the non-profit community organisation Mira to implement the CBD program in Stockholm (including organisation, training and management of CBDs), and to the research group for a robust evaluation via a randomised controlled trial. No funding was made available to conduct an initial pilot study (such as for testing the primary outcomes and recruitment strategies) as the results were required by the funders within a restricted timeframe before the proposed roll-out of the model for all migrant women in need of extra support. A number of antenatal clinics were approached due to the high density of migrant women giving birth according to data from the Swedish Maternal Health Care Register [22]. Women speaking the five languages of the participants were involved in different aspects of the study design as a researcher (AE) or research assistants (NT and others) and commented on the outcome measures and questionnaires, took part in the recruitment procedures and data collection, and continuously informed migrant communities and stakeholders about the study.

### Eligibility criteria

Eligible to participate in the study were nulliparous and multiparous pregnant women between 25 and 35 weeks of gestation who were Arabic-, Polish-, Russian-, Somali- and Tigrinya-speaking, who could not communicate fluently in Swedish, were 18 years or older, had no contra-indications for vaginal birth and consented to access to their birth record data. Somali women are known to carry the highest risk of perinatal morbidity and mortality in comparison with other foreign-born women in Stockholm, Sweden [23] as elsewhere [5,24], communication and language barriers and poorer experiences of labour care [11]. Like Somali-women, many Arabic-speaking and Tigrinya-speaking women (from Eritrea) have migrated to Sweden after traumatic experiences of war and conflict [25]. They, and the Polish and Russian women, constitute a growing group of women of childbearing age in Sweden.

### Recruitment

Women were given information about the study during their usual antenatal care visits between 25 and 35 weeks of gestation at six different antenatal clinics in Stockholm. If they agreed to be contacted, trained bilingual research staff contacted the women by telephone, gave them additional information and asked if they were willing to participate in the study. For those women who agreed to participate, a telephone interview followed in their preferred language (see below).

### Randomisation and blinding

All eligible women who consented to participate and had contributed baseline data were then randomly allocated to the intervention or control group using a computerised randomisation schedule. The randomisation ratio was 1:1, CBD support to standard care, with block sizes of four or six distributed randomly. Allocations were place in sealed, opaque envelopes numbered consecutively for each language group. The bilingual research staff were responsible for opening the envelopes and informing participants of their allocation, after women had responded to the baseline questionnaire. Participants and data collectors could not be blinded in this study, however, analysis was undertaken blinded to group allocation.

### Data collection

Baseline data were collected by the bilingual research assistants by means of a structured questionnaire with closed and open-ended questions. The questions covered the following areas: background (country of origin, first language, knowledge of Swedish, education, marital status, current household members, occupation, reason for migration, length of residence), previous birth(s) in Sweden, planned labour companion, worries, self-rated health and medications. The follow-up questionnaire was completed in a similar way at 6–8 weeks after the birth. The interviews were conducted by the same research assistant and included questions on women’s ratings of care and emotional wellbeing. Full details of the questionnaires are presented elsewhere [13]. Data from medical records were retrieved from The Swedish Pregnancy Register [26]: obstetric background (previous stillbirth and neonatal death, previous mode of birth), present pregnancy (maternal age, parity, BMI at registration, smoking prior to pregnancy, hypertension, diabetes mellitus, number of ANC visits, professional interpreter during ANC visits). The register collects the majority of data directly from electronic medical records and in 2019 covered more than 97.9% of all births in Sweden (www.graviditetsregistret.se).

### Intervention–community-based bilingual doula support

The intervention was supported by a program theory revolving around core values for quality intrapartum care: respect, communication and support [27–29] as outlined in the trial protocol [13], and we hypothesised that the following would improve care and strategies for safe childbirth for migrant women:

- **Improved communication and information support—**to increase mutual understandings about desires and needs, timely apprehension about signs and symptoms for understanding of progress and necessary interventions and strengthen women’s empowerment and rights
- **Common background**–to improve cross-language/culture interactions and empower women to raise their voices in having needs addressed
- **Emotional support and being with the woman—**to reduce anxiety and increase the probability for a normal birth
- **Instrumental support–**to help women manage pain and achieve a normal progress of labour by hands-on comfort measures/physical techniques, appropriate positions during labour and support with energy and fluid intake

The intervention was developed from a model of community-based bilingual doula support meeting all these aspects of care, a model that was already established by a community association, Födelsehuset (Childbirth House) in Gothenburg in the south-west of Sweden. Since 2008 they have provided CBD support during childbirth to around 1500 women who have difficulties communicating in Swedish. Prior to the present study, the non-profit organization Mira replicated the model to provide support for migrant women in the six labour wards in Stockholm, and they recruited, trained and employed the CBDs using similar processes as in Gothenburg. For details see Schytt et al [13]. A total of 23 CBDs were employed for five migrant groups, ensuring the possibility of back-up when needed. The CBD training curriculum covered the physiology of childbirth, strategies for providing effective continuous support in labour and breastfeeding counselling as well as practical strategies for communication assistance between women and midwives/obstetricians and discussion of CBD roles and boundaries. The duration of the training was eight days followed by providing support, under supervision, to three women during labour and birth. CBDs were employed on an hourly basis to enable flexibility in timely provision of support to women in labour.

Women allocated to receive CBD support were contacted by an Arabic-, Polish-, Russian-, Somali-, or Tigrinya-speaking CBD as appropriate, and arrangements made for them to meet twice prior to the birth to get to know each other and discuss the woman’s wishes regarding support in labour and what the CBD could offer. The woman was informed that when labour commenced she should contact the CBD who would assist her to make contact with the labour ward. The CBD would then meet the woman at the hospital and stay with her throughout labour and birth to provide emotional and physical support as well as communication assistance. Having a CBD present during labour did not exclude the possibility of other support people, such as the woman’s partner. After the birth, the woman and CBD met once or twice to follow up on any questions or concerns the woman had regarding the birth and the postpartum period.

### Control–standard intrapartum care

Women allocated to the control arm of the trial received standard intrapartum care as provided at their chosen hospital of birth. This included the support from a midwife and from one assisting nurse who together are responsible for between one and three women in labour. Women are encouraged to bring their partners and/or another support person to be with them during labour and birth. Language interpreting is offered according to the routines of the hospital, however, most interpreting is provided over the phone rather than in person.

### Primary outcomes

Women’s overall ratings of labour care were measured by a single item question taken from the MFMCQ (Migrant Friendly Maternity Care Questionnaire) [30]: ‘In general, were you happy with the healthcare you received?’ with response alternatives dichotomized into ‘Yes, very happy with care’ and ‘Less than very happy’ (Quite happy; Not very happy; No, not happy at all). The survey question was indentified by means of a Delphi consensus process with international perinatal health research experts to be one of a minimum set of questions for capturing migrant womens’ experiences of labour care [30]. Maternal emotional wellbeing was measured by the Edinburgh Postnatal Depression Scale (EPDS), a 10-item self-report scale widely used in research and screening for postpartum depressive symptoms. Each item is scored on a 4-point scale (0–3) and a mean score, as well as a median score, was calculated for each woman [31–33]. For clinical relevance, we also estimated differences in high scores, i.e. above the cut-off 12/13 recommended in Swedish validations [34].

### Secondary outcomes

Different aspects of communication support, overall satisfaction with labour support, whether women would choose the same labour companion for a future labour and birth, and other aspects of care provided by the midwife were assessed (see Table 3). The survey questions and response alternatives are described in the study protocol [13].

### Sample size

To detect an increase in women’s ratings of intrapartum care from an expected 30% saying they were ‘very happy’ among those receiving standard care (based on estimates from population-based studies of migrant women not fluent in the host country language [35] to 53% in those receiving CBD support (equal to Swedish speaking women in a national population based study [36])) with 80% power and an alpha of 0.05, 69 women in each group were needed. To have similar power to detect differences in mean scores on the Edinburgh Postnatal Depression Scale a hypothesised reduction from a mean of 8.0 in the comparison group–similar to that found in studies of migrant women, to 6.0 in the intervention group–similar to that found in Swedish population-based studies [37], 63 women were required in each arm. Allowing for 20% loss to follow-up at the time of data collection with women two months postpartum, we aimed to recruit 174 women.

### Statistical analysis

The cleaned file was locked, the sample for the analysis was determined by coauthors ES, HL and AW and the study arm code was broken on 3 December 2020. A visual check of the comparability of the two groups was made. The intervention group was then compared with the control group testing trial hypotheses. We used intention to treat analyses (ITT) and included all participants who had contributed data at the follow-up interview according to their trial allocation. Table 2 shows that women received various ‘doses’ of the planned intervention, with few participants (15%) receiving all components (homevisit/telephone contact prior to labour, telephone contact after labour had started, CBD support att the labour ward, postpartum follow-up). Given a full *per protocol* analysis was therefore not possible with such a small sample, we instead performed a secondary comparison of primary outcomes for women allocated to the intervention arm who *did* have CBD support during labour and birth (n = 60) with the outcomes for women allocated to SC (n = 64). Analyses were performed using Statistical Package for Social Science version 26 (IBM SPSS Inc, Chicago, IL, USA).

Odds ratios and 95% confidence intervals were estimated using logistic regression for the categorical outcomes (for women’s overall rating of care, specific aspects of care and obstetric outcomes). The distribution of EPDS scores was very skewed. Differences were therefore estimated both for mean values and standard deviations using Students’ t-tests, as presented in the study protocol, and for medians estimated by Mann-Whitney (Mood’s median test) presented with inter-quartile range and range. For clinical relevance, we also estimated differences in high scores, i.e. ≥13 [34]. Missing values on the EPDS scale were very few (baseline n = 8, follow-up n = 4). To confirm the robustness of the analyses and avoid list-wise deletion and potential bias due to missing data in the EPDS, we performed multiple imputations on missing data, assuming that the missing data could be explained by other items on the EPDS scale (missing at random). A total of 20 imputed datasets were created. The results were very similar and analyses without imputations are those presented.

## Results

**Fig 1:**
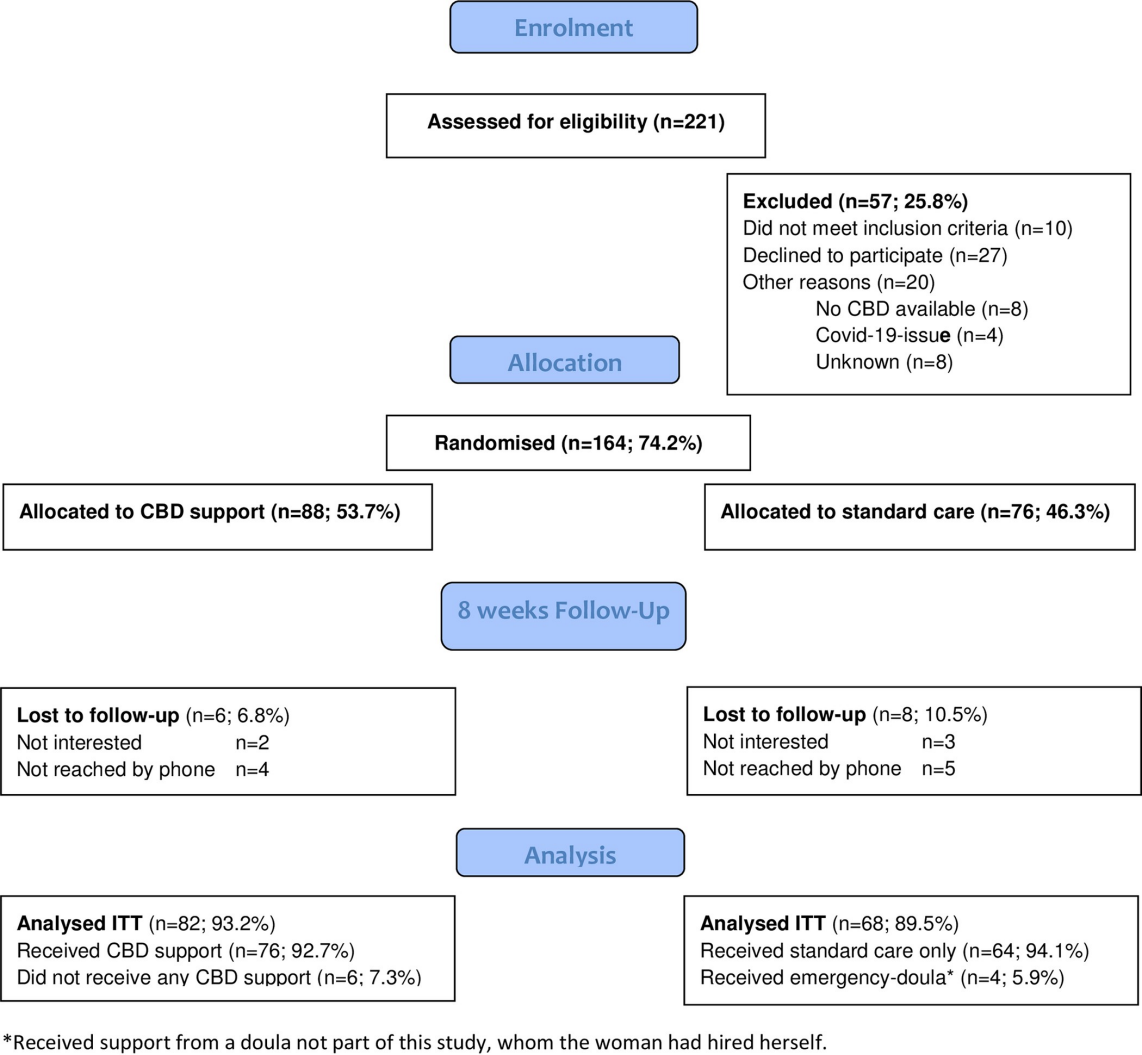
Flowchart of the trial enrolment, allocation, follow-up and analysis.

The flowchart in Fig 1 describes enrolment of the study sample, group allocation, follow-up rates at 8 weeks postpartum and women included in the ITT-analysis. In total, 221 women were assessed for eligibility and 164 (74.2%) participated, after the exclusion of women who did not meet inclusion criteria (n = 10), declined to participate (n = 27) or for other reasons (n = 20). A somewhat higher proportion of women were randomised to the CBD intervention (n = 88; 53.7%) than to SC (n = 76; 46.3%), however, they were evenly distributed by language (CBD: Arabic 55.0%, Somali 52.6%, Tigrinya 51.2%, Russian 55.6%, Polish 56.3%; p = 0.995). The last participant enrolled gave birth on 15 April 2020 and the last follow-up was conducted on 28 15 May 2020, i.e. after the breakout of the COVID-19 pandemic. On 29^th^ April, restrictions were introduced allowing only one support person on the labour wards. Of women allocated to CBD and to SC, 82 (93.2%) and 68 (89.5%) responded to the follow-up questionnaire respectively and are included in the ITT analysis.

Maternal characteristics were largely similar between the CBD and the SC group (Table 1), including age, marital status, reason for migration, previous or present pregnancy, medical conditions, or self-reported health assessments and worries. A somewhat higher proportion of women who received CBD support reported that they did not speak the Swedish language at all or only with difficulties, and also that they had received less professional interpreter support during antenatal visits. Slight differences were observed in education and employment: more women in the CBD group had a university education, fewer were employed and more were currently studying. Given the small numbers in these sub-groups, no adjustment was made for these observed differences.

**Table 1:** Characteristics of women randomised to receive community-based doula support or standard care (n = 164).

	Community-based doula support		Standard Care	
	n = 88 (53.7%)		n = 76 (46.3%)	
	n	%	n	%
**Socio-economics**				
Age, years (mean, SD)	30.3 (5.7)		30.7 (6.3)	
Language				
Arabic	37	42.0	28	36.8
Somali	20	22.7	18	23.7
Tigrinya5	21	23.9	20	26.3
Russian	9	10.2	7	9.2
Polish	1	1.1	3	3.9
Knowledge of Swedish language				
Speak				
Not at all/with difficulties	62	70.5	47	61.8
Well or fluent	26	29.5	29	38.1
Understand				
Not at all/with difficulties	45	51.7	39	51.3
Well or fluent	42	48.2	37	48.7
Education				
None	13	15.3	10	13.2
Elementary school (grade 6–9)	23	27.1	18	23.7
Secondary school (grade 10–12)	20	23.5	24	31.6
University	23	27.1	15	19.7
Other	6	7.1	9	11.8
Marital status				
Married/living with partner/engaged	68	77.3	58	76.3
Single/separated/divorced	20	22.7	18	23.7
Living with				
Husband/partner	62	70.5	55	72.4
Mother/sister/friend	2	2.3	2	2.6
Children only	12	13.6	6	7.9
Alone	12	13.6	13	17.1
Occupation status				
Employed/paid job	10	11.8	16	21.9
Unemployed/looking for a job	5	5.9	4	5.5
Student	5	5.9	6	8.2
Language studies	24	28.2	12	16.4
Home duties/parental leave	38	44.7	33	45.2
Sick leave	3	3.5	2	2.7
**Migration**				
Reason for migration				
Refugee (asylum)	39	45.3	32	42.7
Family ties	44	51.2	39	52.0
Education or work	3	3.5	4	5.3
Length of stay in Sweden (years) (mean, SD)	3.8 (3.5)		4.3 (3.7)	
**Obstetric background**				
Neonatal death*	1	1.3	0	0
At least one previous birth in Sweden*	35	63.6	31	70.5
Previous caesarean section*	7	8.6	6	8.2
**Present pregnancy and health**				
Parity				
Nulliparous	33	37.5	32	42.1
Multiparous	55	62.5	44	57.9
BMI (mean (SD)	25.1 (5.3)		25.9 (4.4)	
Smoking 3 months before pregnancy	4	5.1	3	4.2
Hypertension	1	1.3	0	0
Diabetes mellitus	1	1.3	0	0
Number of antenatal care visits (mean, SD)	7.9 (3.0)		8.6 (2.6)	
**Self-reported assessments and expectations at recruitment**				
Poor self-rated health (neither good nor bad/bad/very bad)	27	31.0	21	27.6
Self-reported medical conditions or physical health issues	11	12.6	7	9.2
Self-reported current medications	14	16.3	13	17.3
Have someone to accompany for labour	63	73.3	55	73.3
Number of worries for the upcoming labour and birth (mean, SD)	2.6 (3.1)		2.6 (3.0)	

*Multiparous women only in the analyses.

Of women randomised to CBD support, the vast majority (n = 76; 92.7%) received at least one component of the intervention, however, few women (n = 13; 15.9%) received all possible components. In total, 72 (87.8%) women received home visit(s) or telephone call(s) prior to labour to establish contact with the CBD, 21 (25.6%) received phone support at home when the labour started, 60 (73.2%) received CBD support on the labour ward and 52 (63.4%) had a home visit or telephone call for follow-up. The CBD was known prior to labour in nearly all women who were supported on the labour ward (n = 58; 96.7%). Reasons for not having a CBD on the labour ward, despite being allocated one, were as follows: miscommunication (n = 6), the woman’s own wish (n = 5), CBD unavailable (n = 4) or not there in time (n = 4), doulas not allowed due to the COVID-19 pandemic (n = 1) and unknown reasons (n = 2). Of women randomised to standard care, 4 (5.9%) received support from a doula not part of this study, whom she had hired herself.

### Primary outcomes

Women’s emotional wellbeing did not differ between the groups (Table 2). Mean scores on the EPDS were similar at baseline (CBD 7.34 vs SC 6.71; mean difference 0.63; CI 95% -2.56–1.30) and at the postpartum follow-up (CBD 4.76 vs SC 3.44; mean difference 1.32; CI 95% -2.78–0.13). EPDS medians were also estimated due to the skewed distribution and were similar at baseline (CBD 6 vs SC 6; median difference 0; p = 0.953) and at follow-up (CBD 4 vs SC 3; median difference 1; p = 0.147). Finally, the proportion of women scoring ≥13 on the EPDS was high in both groups at baseline (CBD 21.1% vs SC 13.6%; OR 1.69; CI 95% 0.65–3.87) and similarly lower at follow-up (CBD 8.8% vs SC 1.5%; OR 6.23; CI 95% 0.75–52.02). Analysis of data with imputed missing values on the EPDS did not change the result (not shown).

**Table 2:** Women’s wellbeing measured by the EPDS; mean scores (as in protocol) and median scores (due to highly scewed results).

	Community-based doula				Standard Care				Comparisons between groups			
	Mean scores											
	Mean	Range	SD	Std.Error mean	Mean	Range	SD	Std.Error mean	Mean difference	Std.Error mean	95% CI of the difference	p
EPDS at inclusion	7.34	0–25	5.99	0.687	6.71	0–23	5.55	0.683	0.630	0.974	- 2.56–1.30	0.519
EPDS 2 months postpartum	4.76	0–23	5.01	0.560	3.44	0–14	3.61	0.445	1.32	0.737	-2.78–0.13	0.075
	**Median scores**											
	**Median**	**Range**	**IQR**		**Median**	**Range**	**IQR**		**Median difference**			**p**
EPDS at inclusion	6	0–25	7		6	0–23	8.25		0			0.953
EPDS 2 months postpartum	4	0–23	6		3	0–14	4		1			0.147

**Fig 2:**
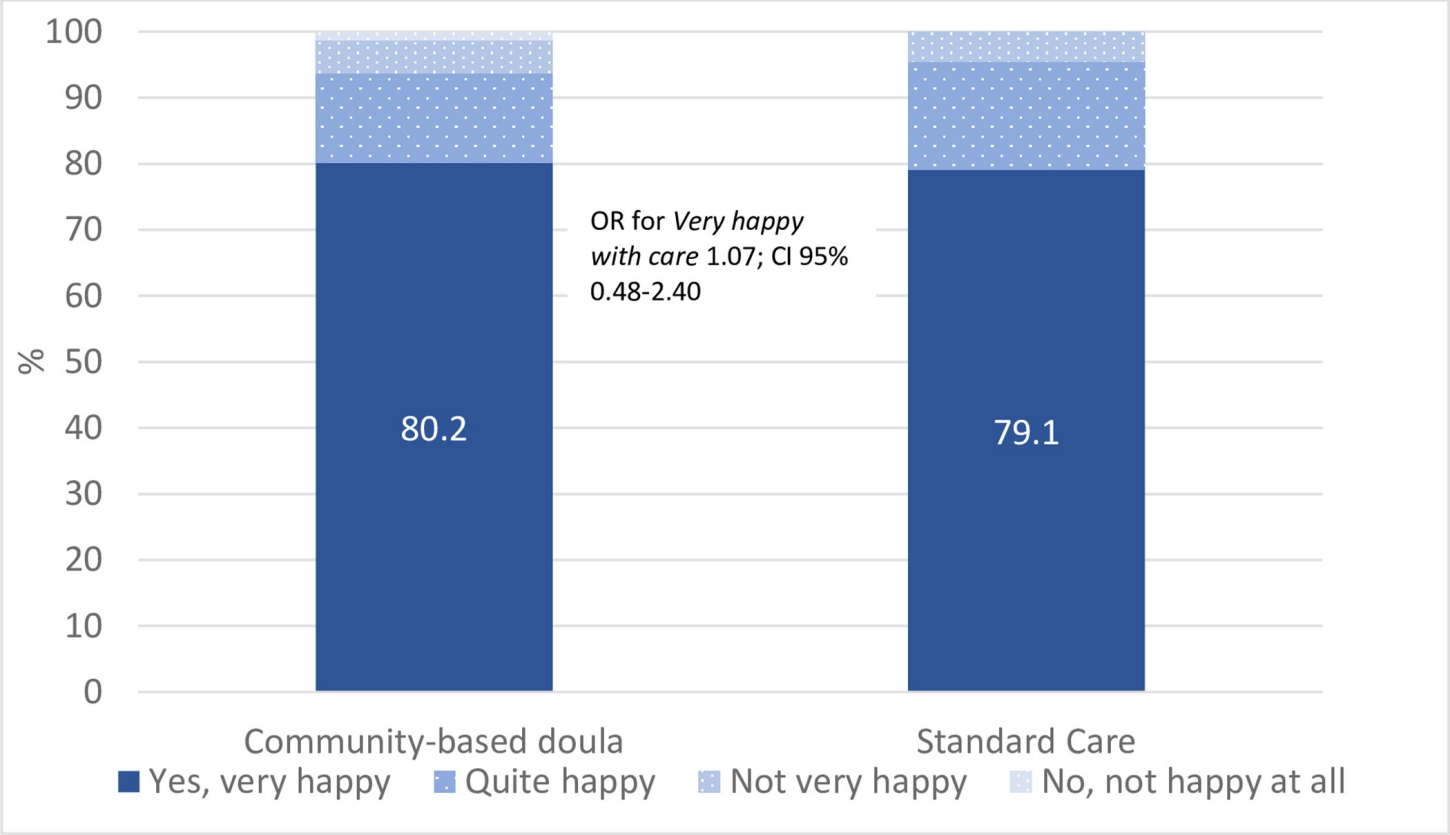
Overall ratings of care in women with community based doula support and women with standard care.

Women’s overall ratings of care two months after the birth were also similar between the groups (Fig 2). Of the women allocated to receive CBD support, 80.2% were very happy with care, as were 79.1% of women who received SC (OR 1.07; CI 95% 0.48–2.40).

### Secondary outcomes

Women’s ratings of communication, labour support and other aspects of care did not differ significantly between the groups (Table 3), although on most items the proportion of women reporting positive experiences was greater in the CBD group. Regardless of allocation, very few women (CBD 5.5% vs SC 10.5%; OR 0.49 CI 95% 0.31–1.84) were provided a professional interpreter during labour and birth. In spite of this, nearly all women were satisfied with the linguistic support they had received (CBD 94.5% vs SC 92.6%; OR 1.38 CI 95% 0.33–5.78). Of note, a higher proportion of women in the CBD group reported that they understood the information provided by health care professionals, than in the SC group, although the difference was not statistically significant (72.8% vs 59.1%; OR 1.86; CI 95% 0.93–3.71). Similarly, a higher proportion of women with CBD support had felt comfortable asking questions (72.8% vs 58.2%; OR 1.93; CI 95% 0.97–3.84). Regardless of allocation, the vast majority of women said that they had received sufficient information about pain, pharmacological pain relief, the health of the baby and the partner’s role during labour and birth, but only half were asked about their own preferences. Table 3 also shows that the vast majority of women were very happy with all the support received during labour and birth and had positive experiences of the care provided by the midwife (Table 3).

**Table 3:** Women’s assessments of communication, support and other aspects of care in relation to intervention.

	Community-based doula support		Standard Care		OR	CI 95%
	n	%	n	%		
**Communication**						
Provided a professional interpreter (yes *vs* no)	4	5.5	6	10.5	0.49	0.31–1.84
Satisfied with linguistic support (yes *vs* no)	69	94.5	50	92.6	1.38	0.33–5.78
Understood the information provided by staff (Always *vs* Sometimes, Rarely, Never, Don’t know)	59	72.8	39	59.1	1.86	0.93–3.71
Felt comfortable asking questions (Always *vs* Sometimes, Rarely, Never)	59	72.8	39	58.2	1.93	0.97–3.84
Received sufficient Information regarding						
How to manage labour pain	74	91.4	60	90.9	1.06	0.34–3.31
Pharmacological pain relief	69	86.3	56	87.5	0.90	0.34–2.38
Health of the baby	71	88.8	57	87.7	1.11	0.40–3.05
Partner’s role	65	81.3	53	82.8	0.90	0.38–2.12
Was asked about own preferences (if had not expressed them herself) (yes *vs* no)	43	57.3	37	59.7	0.91	0.46–1.80
Was asked to do something she did not want to do (yes *vs* no)	6	7.4	3	4.5	1.71	0.41–7.10
Felt discriminated against (yes *vs* no)	5	6.2	3	4.5	1.38	0.32–6.01
**Labour support**						
Happy with all support received during childbirth (very happy *vs* quite happy, not very happy, not happy at all)	63	78.8	47	70.1	1.58	0.75–3.34
Would choose the same labour companion if giving birth again (yes *vs* no)	72	94.7	58	92.1	1.55	0.40–6.04
**Other aspects of care provided by the midwife** (always vs sometimes, rarely, never)						
I felt welcomed by the midwife	76	93.8	59	86.8	2.32	0.74–7.29
She was respectful	76	93.8	66	97.1	0.46	0.09–2.45
She spent enough time providing explanations	67	82.7	56	82.4	1.03	0.44–2.40
Decisions were made without my wishes being taken into account	10	12.5	8	11.8	1.07	0.40–2.89
She kept me informed about what was happening	72	90.0	55	82.1	1.96	0.75–5.13
I felt my worries were taken seriously	66	83.5	51	76.1	1.59	0.70–3.61
I felt confident that she was skilled in medical issues	73	90.1	62	93.9	0.59	0.17–2.05

There were no statistically significant differences between the groups on any of the obstetric outcomes (Table 4). Women had similar rates of induction of labour, epidural analgesia, episiotomy, perineal injury (degree III-IV) and blood loss. Fewer women in the CBD group experienced vacuum extraction (3.8% vs 6.2%), emergency caesarean (10.3% vs 13.8%) and elective caesarean (1.3% vs 4.6%) though none of these differences was statistically significant (not shown). While a higher proportion of women in the CBD group had a non-instrumental vaginal birth (84.6%) compared with women in the SC group (75.4%), this was not statistically significant (OR 1.80, CI 95% 0.80–4.14). Overall the odds of a caesarean birth were lower in the CBD group, but this failed to reach statistical significance (OR 0.58; CI 95% 0.23–1.47), and similarly for all operative births (OR 0.56; CI 95% 0.24–1.28). Infant outcomes (Apgar <7, perinatal death, breastfeeding at discharge and 2 months postpartum) were also similar between the groups. Finally, the length of time (hours) from admission to the labour ward up to the birth (CBD mean 12.2, SD 12.0; SC mean 12.2, SD 14.8; mean diff = 0.02; CI 95% -4.41–4.46) and from the birth to discharge (CBD mean 73.9, SD 33.9; SC mean 67.4, SD 27.6; mean diff 6.53; Ci 95% -3.96–17.01) did not differ between the groups.

**Table 4:** Obstetric outcomes for women with CBD support or standard care during labour and birth (n = 143).

	Community-based doula support		Standard Care		OR	CI 95%
	n = 78		n = 65			
	n	%	n	%		
**Maternal outcomes**						
Induction of labour	9	11.0	8	11.0	0.93	0.34–2.56
Epidural analgesia	31	39.7	24	36.9	1.13	0.57–2.22
Episiotomy	5	6.4	1	1.5	4.38	0.50–38.51
Caesarean section (vaginal birth is reference)	9	11.5	12	18.5	0.58	0.23–1.47
Operative birth (non-instrumental vaginal is reference)	12	15.4	16	24.6	0.56	0.24–1.28
Perineal injury degree III-IV	3	3.9	3	4.6	0.84	0.16–4.30
Blood loss (ml) (mean, SD)	454 (324)		439 (263)		15.38*	-98.85–94.89*
**Infant outcomes**						
Apgar score < 7 at 5 minutes	1	1.3	1	1.6	0.84	0.05–13.7
Perinatal death	0		0			
Breastfeeding at discharge	71	86.6	61	89.7	0.74	0.27–2.03
Breastfeeding 2 months postpartum	40	48.8	37	54.4	0.80	0.42–1.52

* Students’ t-test with 95% CI for mean differences.

A secondary comparison of the primary outcomes for women allocated to the intervention arm who actually *received* CBD support during labour and birth (n = 60) with the outcomes for women allocated to SC (n = 64) showed no differences between groups; neither their wellbeing (EPDS mean scores: CBD 5.1 vs SC 3.5; mean diff = 1.6; 95% CI -0.02–3.16) nor their overall ratings of care (very happy with care: CBD 78.0% vs SC v 81.0%; OR 0.83 CI 95% 0.35–2.01).

## Discussion

The two hypotheses that community-based bilingual doula support would increase migrant women’s overall ratings of care for labour and birth and improve their postnatal emotional wellbeing were not confirmed. Regardless of allocation, women reported high satisfaction with care and good postnatal emotional wellbeing. The findings do suggest though, that CBDs have the potential to improve communication between the labouring woman and staff. Our study was not powered to detect differences in mode of birth outcomes, but the lowered odds found for caesarean section and instrumental birth in the CBD group, while not statistically significant, do warrant larger, adequately powered studies.

A strength of the study was the pragmatic trial design, i.e. the intervention was evaluated for effectiveness in the context of everyday maternity care settings in order to maximize applicability and generalizability [38,39]. Women speaking five different languages representing the most common migrant populations in Sweden and those with particularly high risk for adverse outcomes were recruited from six different ANC clinics and they gave birth in five hospitals in Region Stockholm. Only 27 women (12.2%) declined to participate and 9.8% did not remain to follow-up, compared with an anticipated 20%. We believe that the involvement of bilingual research assistants contributed greatly to the high participation rate, enabling accessible and appropriate information about the study to be given, and increasing women’s sense of safety in taking part. Recruitment was terminated when 164 women had contributed baseline data and just as the Covid-19 pandemic broke out, which restricted support people in the labour room to one, preferencing the woman’s partner. According to the power analyses, we received data from a sufficient number of women for the ITT-analyses for one of the primary outcome measurements *women’s emotional wellbeing* as measured by the EPDS (63 women needed). For *overall ratings of care*, we analysed a sufficient number of women in the CBD group and 68 (69 needed) in the SC group. Adding one ‘dummy’ participant to the SC group in two separate analyses, with the highest or lowest possible value respectively, did not change the results (available on request). Further, in this study, 80% of the migrant women were ‘very happy’ with care, also in the comparison group, and very few (5%) were not at all happy with their care. This differs greatly from the assumptions for the power analysis that around 30% would say they were ‘very happy’ with care among those receiving standard care, based on past studies of migrant women not fluent in the host country language [35] in comparison with the level in Swedish women, i.e. 53% in a Swedish national population based study [36].

Very few women, (n = 13; 7.9%), received the planned package of the intervention in its entirety, even though most (73%) received CBD support on the labour ward, likely the most important component. Some of the reasons for the CBD not being present for labour may not however, be preventable. These include a short labour that reduces a CBD’s chance of reaching the labour ward in time or that women (or their partners) change their minds about having CBD support at the time of labour onset. It is important to remember however, that the CBD model had just been implemented in Stockholm, and much was being learned by everyone involved about how best to organise and support the CBDs. A number of teething problems with the organisation of the model were identified when data from interviews with midwives and obstetricians on the same labour wards were analysed. Some areas of improvement were identified, such as aspects of communication support and the organisation of back-up for CBDs [19].

CBD support did not improve women’s emotional wellbeing 6–8 weeks after the birth as measured by the EPDS. At inclusion, a high proportion of women had above the cut-off 12/13, i.e. 21.1% and 13.6% in the CBD and SC groups respectively, and higher than in a Norwegian population-based study of a multi-ethnic population [40]. One might assume that the extra support provided by the CBD would be attractive to women with depressive symtoms [41] and a previous study showed that vulnerable women are more likely to accept a CBD when offered, than healthy, more socio-economically advantaged women [19]. The EPDS scores at follow-up were low in both groups whether comparing means, medians or proportions of women with scores above the recommended cut-off [34], and much lower than previously reported in Swedish population-based studies [37]. We had hypothesised that women’s wellbeing would improve if they were better supported during childbirth, [42] and that reduced anxiety and risk for operative birth would improve their birth experiences [41,43,44]. A Hawthorne effect in both groups cannot be dismissed, as all women interacted with a bilingual research assistant from their own culture who actively listened to their experiences of pregnancy, labour and birth.

Four out of five women, were ‘very happy’ with care regardless of allocation, a positive but unexpected finding. In a recent national report, the overall proportion of women who rated their experience with care as ‘very positive’ corresponding to 8 or above on a scale from 0–10, increased slightly from 70 to 73 percent in Sweden between 2015 and 2020 and the numbers did not differ between women born in Sweden or in migrant women in general [45]. Although there is no consensus about what patient satisfaction encompasses, Risser already in 1975 described patient satisfaction as an attitude that reflects the congruence of what a patient expects and the care received [46]. One interpretation of the highly positive rating of care found in the current study is that women from specifically low and middle-income countries in North and Sub-Saharan Africa, Middle East and Eastern Europe may have low expectations of labour care and support provided by public institutions based on their previous experiences. Poor quality of public maternity care, lack of labour support and disrespectful treatment has been reported for instance from North and Sub-Saharan Africa [47–49]. The CBD-intervention was designed as a woman-centred care model, based on a program theory revolving around the core values respect, communication and support, to optimize women’s experience of childbirth through a holistic approach [13]. This may have been neglected in women’s previous labour and births, or those of family members, affecting their expectations [50].

There was no evidence in our trial that obstetric outcomes were impacted by the provision of a CBD, but the small size of the trial may be the reason. The odds for both a caesarean section and an operative birth (caesareans and vacuum extractions) were halved in the CBD arm compared with standard care, but were not statistically significant. A register-based cohort study [51] evaluating the CBD program in Göteborg, Sweden, showed similar rates of caesareans and instrumental vaginal births in CBD-supported migrant women as in migrant women without such support, but there was a higher prevalence of risk factors for adverse outcomes in the CBD group. Given this risk imbalance between the two migrant cohorts, similar levels of caesarean section and overall operative birth could be interpreted as a positive result for the impact of CBD support. Findings from both studies point to the need for adequately powered trials to evaluate the effectiveness of CBD support for reducing caesarean section and operative birth. The findings of our trial can also inform the choice of primary outcomes and power calculations for future trials. To detect a statistically significant and also clinically relevant reduction in caesarean section from 19% to 12% (which was the case in our study), 836 women are needed, 418 in each arm. Additionally, to increase the rates of non-instrumental vaginals births, from 75% to 85%, 500 women in total would be needed.

Although the study did not demonstrate any statistically significant effects on the primary outcomes, the results overall indicate that it would be premature to dismiss CBD support as a promising intervention to improve specific components of care during labour and birth for migrant women in Sweden. For most of the more detailed aspects of care, although group differences did not reach statistical significance, women in the CBD group were more likely to be positive about their care. More women assisted by a CBD reported that they understood information provided by staff and had felt more comfortable asking questions, than women in the SC group. Moreover, qualitative studies conducted as part of the process evaluation of this trial of a complex intervention showed that the hypothezised mechanisms appear to have occurred. The midwives and obstetricians who had worked alongside the CBDs noted in interviews that the CBDs had improved communication, given both the language support provided to women and their shared backgrounds, and they had contributed valuable emotional and instrumental support [19]. Women’s assessments of communication with staff during labour and birth may be an even more important outcome measurement when investigating quality of care than the concept of overall satisfaction with care [52,53]. Additionally, indicating a positive interaction between the intervention and the health care system [39], the midwives and obstetricians welcomed the CBDs as a new actor in the team around the labouring woman as the CBDs had lessened their workload and contributed to safer labours and births [19]. The CBDs themselves reported how the close relationship established with women during pregnancy had enabled more woman-centred care during labour and birth, with continuous linguistic and cultural support as major components [54]. Additionally, the fact that migrant women were very interested to take part in the study indicates a positive attitude to bilingual doula support [14].

The results indicate however, that the communication components in the intervention could be further developed and evaluated. We had anticipated that CBD support would empower women through improved communication and support from a peer from their own community and culture [55,56]. While most women said that they were kept informed about what happened, 10% in both groups said that decisions were made without their wishes being taken into account and almost half were not asked about their own preferences. Additionally, more than a quarter in the CBD group (compared with 40% in SC) said they did not understand the information provided or did not feel comfortable asking questions. In the interviews, midwives and obestetricans reported that even though the presence of a CBD significantly improved communication in the labour room, some CBDs had language difficulties themselves and the staff needed to simplify information and instructions to accommodate this or use a professional interpeter [19]. While it was never intended that the bilingual doulas would replace professional interpreters, who should always be called in when needed, it may be desirable in future to increase the requirement for adequate Swedish language skills when doulas are recruited.

## Conclusion

Community-based bilingual doula support neither improved migrant women’s overall ratings of care for labour and birth, nor their emotional well-being postpartum. The results indicate however, that CBD support has the potential to improve communication between migrant women and their caregivers. Improvements to the Swedish model of CBD support should focus on these communication aspects. Future studies should be powered to provide robust assessment of the effectiveness of CBD support in safely reducing caesarean section and increasing non-instrumental vaginal birth in migrant women.

## Supporting information

S1 Checklist (PDF)
